# [Retracted] Silencing of GP73 inhibits invasion and metastasis via suppression of epithelial-mesenchymal transition in hepatocellular carcinoma

**DOI:** 10.3892/or.2025.8943

**Published:** 2025-07-07

**Authors:** Ying Yang, Qiang Liu, Hua Zhang, Huarong Zhao, Ruin Mao, Zhipeng Li, Sha Ya, Chunli Jia, Yongxing Bao

Oncol Rep 37: 1182–1188, 2017; DOI: 10.3892/or.2017.5351

Following the publication of this paper, it was drawn to the Editor's attention by a concerned reader that Fig. 3A (showing the results of scratch-wound assay experiments) and Fig. 4C (showing the results of cell morphological experiments) both contained internally a pair of data panels containing duplicated data. Moreover, four of the six data panels shown in Fig. 3B (showing the results of Transwell migration assay experiments), contained either matching or overlapping data panels. In addition, the western blot data in Fig. 4A selected to show the Vimentin protein bands for the MHCC97H cell line and the Snail protein bands for the Bel-7404 cell line were seemingly identical. Finally, it was noted that certain of the western blot data in Fig. 4A of the above paper were also apparently included in Figs. 2A and 3A of another paper published by the same group in the journal *Carcinogenesis*.

Owing to the number of identified instances of data duplication in this paper, the Editor of *Oncology Reports* has decided that it should be retracted from the Journal on account of a lack of confidence in the presented data. The authors were asked for an explanation to account for these concerns, but the Editorial Office did not receive a reply. The Editor apologizes to the readership for any inconvenience caused.

